# Metal-Coordinated Dynamics and Viscoelastic Properties of Double-Network Hydrogels

**DOI:** 10.3390/gels9020145

**Published:** 2023-02-09

**Authors:** Shilei Zhu, Yan Wang, Zhe Wang, Lin Chen, Fengbo Zhu, Yanan Ye, Yong Zheng, Wenwen Yu, Qiang Zheng

**Affiliations:** 1 College of Physics, Taiyuan University of Technology, Taiyuan 030024, China; 2 College of Materials Science & Engineering, Taiyuan University of Technology, Taiyuan 030024, China; 3 Shanxi-Zheda Institute of Advanced Materials and Chemical Engineering, Taiyuan 030024, China; 4 Institute for Chemical Reaction Design and Discovery, Hokkaido University, Sapporo 001-0021, Japan; 5 Ministry of Education Key Laboratory of Macromolecular Synthesis and Functionalization, Department of Polymer Science and Engineering, Zhejiang University, Hangzhou 310027, China

**Keywords:** metal-coordinated dynamics, viscoelastic properties, double-network gels

## Abstract

Biological soft tissues are intrinsically viscoelastic materials which play a significant role in affecting the activity of cells. As potential artificial alternatives, double-network (DN) gels, however, are pure elastic and mechanically time independent. The viscoelasticization of DN gels is an urgent challenge in enabling DN gels to be used for advanced development of biomaterial applications. Herein, we demonstrate a simple approach to regulate the viscoelasticity of tough double-network (DN) hydrogels by forming sulfonate–metal coordination. Owing to the dynamic nature of the coordination bonds, the resultant hydrogels possess highly viscoelastic, mechanical time-dependent, and self-recovery properties. Rheological measurements are performed to investigate the linear dynamic mechanical behavior at small strains. The tensile tests and cyclic tensile tests are also systematically performed to evaluate the rate-dependent large deformation mechanical behaviors and energy dissipation behaviors of various ion-loaded DN hydrogels. It has been revealed based on the systematic analysis that robust strong sulfonate–Zr^4+^ coordination interactions not only serve as dynamic crosslinks imparting viscoelastic rate-dependent mechanical performances, but also strongly affect the relative strength of the first PAMPS network, thereby increasing the yielding stress σ_y_ and the fracture stress at break σ_b_ and reducing the stretch ratio at break λ_b_. It is envisioned that the viscoelasticization of DN gels enables versatile applications in the biomedical and engineering fields.

## 1. Introduction

Hydrogels have recently emerged as promising candidates for use in artificial cartilage engineering because they possess cartilage tissue-like features such as ease of fabrication, toughness, surface smoothness, biocompatibility, and appropriate biochemical properties. However, conventional hydrogels are usually too mechanically weak and brittle to sustain external loads, owing to the inhomogeneous and amorphous structure of the polymer network; however, the natural cartilage is mechanically strong and tough, and can even sustain compressive stress as great as tens of MPa [1,2]. These huge gaps in the mechanical properties between conventional synthetic weak hydrogels and strong load-bearing biological tissues significantly impede the advanced functional applications of these hydrogels as biomaterials. 

To achieve better mechanical properties for conventionally weak hydrogels, in the last few decades, many great efforts have been made in fabricating strong and tough hydrogels through chemical modification methods [3,4,5,6,7] (e.g., by introducing sacrificial bonds [8,9,10,11] and making a homogeneous polymer network structure [4,6]) and by incorporating reinforcing nanofillers [12,13,14,15,16,17]. Among these great efforts, the double-network (DN) approach stands out as an extraordinarily effective method for developing strong and tough materials, and the “sacrificial bonds” mechanism has been proved to efficiently increase energy dissipation for improving toughness and strength synchronously [5,10]. A hydrogel with DN structure showing extremely enhanced mechanical strength and toughness is designed to be composed of two types of interpenetrating polymer network with contrasting natures, i.e., a densely crosslinked stiff and brittle polyelectrolyte first network, and a loosely crosslinked soft and ductile neutral second network [10]. Upon deformation, the brittle first network can break into fragments and dissipate a large amount of energy, while the stretchable second network can keep its integrity without causing catastrophic failure of the whole hydrogel [10].

Previous studies have shown that a conventional DN gel composed of poly(2-acrylamido-2-methyl propanesulfonic acid) (PAMPS) as the brittle first network and poly(N,N-dimethylacrylamide) (PDMA) or polyacrylamide (PAAm) as the ductile second network is a promising biomaterial for biological tissues (e.g., artificial cartilages) [18,19]. It has been demonstrated that DN gel has excellent biocompatibility and a low degree of toxicity in vivo, high compressive strength, extremely low friction, and low wear production in vitro and in ex vivo friction tests [20,21,22,23,24]. On the other hand, natural biological tissues are intrinsically viscoelastic materials [25,26], and recent studies have shown that the viscoelasticity, especially the time-dependent stiffness, plays a significant role in affecting the activity of cells [27,28,29,30,31]. Unlike natural biological tissues, traditional DN hydrogels are rate independent and elastic. Thus, achieving their viscoelastic transformation is a pressing issue in advancing conventional DN gel for biomaterial applications, and can help deepen our understanding of the regulated viscoelasticity and activity of biological tissue cells. Introducing dynamic bonds is a common strategy to impart viscoelasticity to materials [9,32,33,34]. The dynamic bonds include many types of physical interactions, such as van der Waals interactions [35,36], π−π stacking interactions [37,38], hydrogen-bonding interactions [33,39], ionic interactions [9,40], metal−ligand interactions [32,41,42], and so on.

Taking advantage of sulfonate groups in the PAMPS first network, herein, we demonstrate a simple approach to regulate the viscoelasticity of traditional DN hydrogels. The traditionally rate-independent elastic PAMPS/PAAm DN hydrogels were tailored to exhibit rate-dependent viscoelastic mechanical responses by forming sulfonate–M^n+^ coordination complexes that act as dynamic crosslinks to confer viscoelasticity [41]. The PAMPS first network, with plenty of sulfonate (–SO_3_^−^) functional groups, provides functional sites for forming metal–coordination complexes [41,42]. Here, the Zr^4+^ was chosen as the model ion, and Fe^3+^ and Na^+^ ions were chosen for comparisons. We first performed rheological measurements to investigate the linear dynamic mechanical behavior at small strain. Further tensile tests and cyclic tensile tests were then systematically conducted to evaluate the rate-dependent large deformation mechanical behaviors and energy dissipation behaviors for various ion-loaded DN hydrogels. The effect of robust sulfonate–Zr^4+^ coordination interactions on the rate-dependent mechanical performances is carefully detailed in the discussion section. This work offers a simple strategy for tuning the viscoelasticity of conventional elastic DN hydrogels, which could expand the application of DN hydrogels as biological artificial tissues. 

## 2. Results and Discussion

### 2.1. Hydrogel Fabrication Process

**Figure 1:**
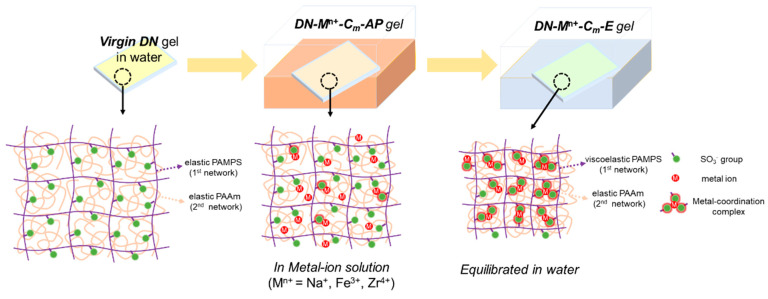
Schematics of design and transformation of the virgin elastic DN hydrogels to viscoelastic materials via metal-coordination complex. As-prepared DN hydrogels in metal–ion solutions are denoted as “DN−M^n+^−C_m_−AP”, while the DN hydrogels equilibrated in water afterward are denoted as “DN−M^n+^−C_m_−E”, where the notations “AP” and “E” mean “as-prepared” and “equilibrated”, respectively, and C_m_ (M) represents the metal–ion concentration in solution. The metal–ions used in this work are Na^+^, Fe^3+,^ and Zr^4+^.

The conventional PAMPS/PAAm DN hydrogels [43] were selected as model systems in this work due to the presence of plenty of sulfonate (–SO_3_^−^) functional groups in the PAMPS of the first network providing functional sites for the formation of metal coordination, and the extensive research on their use as artificial tissue materials in previous works [18,19,20,21,22,23,24]. Here, the multivalent Zr^4+^ and Fe^3+^ were chosen as metal ions for their coordination abilities with sulfonates, as reported in previous studies [41,42]; conversely, the monovalent Na^+^ ion, which lacks coordination ability with sulfonates, was chosen as a reference. Figure 1 illustrates the fabrication procedure of ion-coordinated viscoelastic DN hydrogels from virgin elastic DN hydrogels through immersion in metal–ion solutions and subsequent dialysis in water. For simplification, the DN hydrogels equilibrated in metal–ion solution (in the as-prepared state) and the hydrogels subsequently equilibrated in water are denoted as “DN−M^n+^−C_m_−AP” and “DN−M^n+^−C_m_−E” hydrogels, respectively, where M*^n^*^+^ represents Zr^4+^, Fe^3+^ and Na^+^ ions in this work, and C_m_ (M) represents the molar concentration of metal–ion in solution. In the process of transformation from DN−M^n+^−C_m_−AP gel to DN−M^n+^−C_m_−E gel, counter ions were dialyzed out from the network, and more dynamic coordination bonds were formed, giving the latter a stronger viscoelasticity. By forming robust sulfonate–Zr^4+^ or sulfonate–Fe^3+^ coordination complexes, the purely elastic PAMPS network will be transformed into a viscoelastic one, imparting rate-dependency and viscoelasticity to the DN hydrogels. For the Zr^4+^ and Fe^3+^ ions-loaded DN hydrogels, the concentration of metal ion solution C_m_ is fixed at a relatively small concentration of 0.1 M, while for the Na^+^ ion-loaded DN hydrogels, the concentration of metal ion solution C_m_ is fixed at a high concentration of 1.0 M to demonstrate the ineffective effect of Na^+^ ions. Note that the monomer concentration of AMPS monomer for the PAMPS network in the final DN hydrogels can be estimated as 0.05 M, which means that the effective concentration of sulfonate (–SO_3_^−^) functional groups is ~0.05 M. 

### 2.2. Linear Dynamic Mechanical Behavior at Small Strain

**Figure 2:**
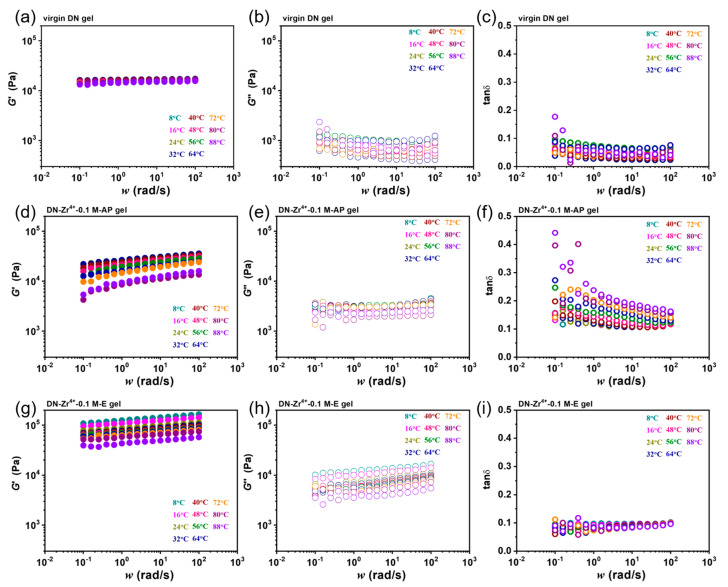
Linear dynamic mechanical behavior of virgin DN, DN−Zr^4+^−0.1 M−AP and DN−Zr^4+^−0.1 M−E hydrogels. Frequency dependence of (**a**) storage moduli G′, (**b**) loss moduli G″ and (**c**) loss factor tan *δ* for virgin DN hydrogels. Frequency dependence of (**d**) storage moduli G′, (**e**) loss moduli G″ and (**f**) loss factor tan *δ* for DN−Zr^4+^−0.1 M−AP; frequency dependence of (**g**) storage moduli G′, (**h**) loss moduli G″ and (**i**) loss factor tan *δ* for DN−Zr^4+^−0.1 M−E hydrogels.

Since the dynamic metal coordination bonds are sensitive to temperature, we chose frequency sweep rheological measurements at different temperatures to verify our proposed approach. Here, Zr^4+^−coordinated DN gels were selected as a typical example. Figure 2 depicts the frequency (ω) sweep of G′, G″, and tan*δ* from 0.1 to 100 rad/s at various temperatures ranging from 8 °C to 88 °C for the DN−Zr^4+^−0.1 M−AP and DN−Zr^4+^−0.1 M−E hydrogels. For the virgin DN gel, the G′ is frequency independent in the full frequency range. Furthermore, with an increase in temperature, the G′ shows negligible change, indicating that the virgin DN gel is pure elastic, and time–temperature superposition fails for such kinds of material. Different from the virgin DN gel, the G′ for the DN−Zr^4+^−0.1 M−AP and DN−Zr^4+^−0.1 M−E hydrogels show strong frequency-dependent and temperature-dependent behaviors, indicating that the dynamic metal coordination bonds were successfully introduced into the DN gels. Besides, it is interestingly found that compared to the DN−Zr^4+^−0.1 M−AP hydrogel, the DN−Zr^4+^−0.1 M−E hydrogel shows a much higher G′ at the same frequency and temperature. The lower G′ for the DN−Zr^4+^−0.1 M−AP hydrogel could be attributed to the excess of Zr^4+^ ions in DN−Zr^4+^−0.1 M−AP hydrogels, which weakens the sulfonate–Zr^4+^ coordination bonds in the first PAMPS network; by moving the gels to DI water, the excessive free Zr^4+^ ions will be dialyzed out from the gel to achieve a new equilibrium, helping to form stronger sulfonate–Zr^4+^ coordination in the gels. To confirm the formation of metal coordination, we further studied the rheological behaviors of DN−Na^+^−1.0 M−AP and DN−Na^+^−1.0 M−E hydrogels, where the Na^+^ lacks the coordination ability with sulfonate. As shown in Figure S1, both gels show frequency and temperature independence similar to that of virgin gels. The above comparison demonstrated that only the metal ions with coordination ability with sulfonate can be used to achieve the viscoelasticization of DN gels.

**Figure 3:**
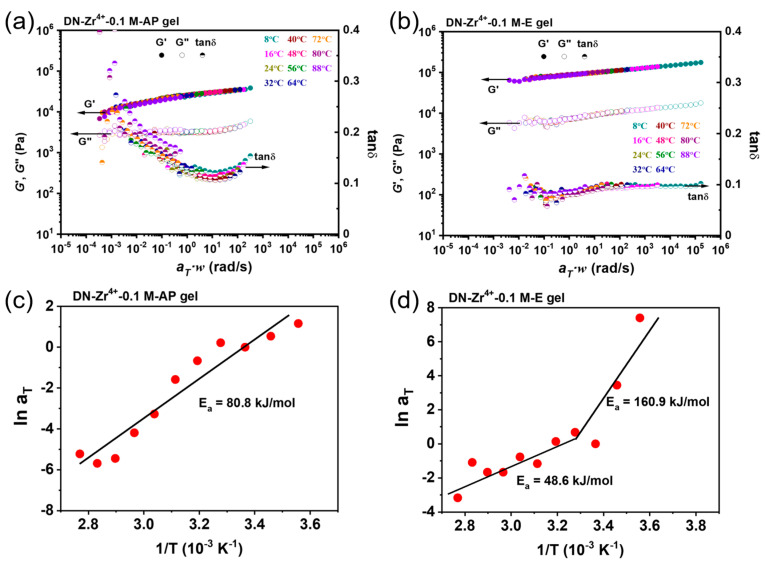
Master curves of the storage modulus G′, loss modulus G″, and loss factor tanδ of DN−Zr^4+^−0.1 M−AP hydrogels (**a**) and DN−Zr^4+^−0.1 M−E hydrogels (**b**). The measurements were performed from 0.1 to 100 rad/s at a shear strain of 0.2%, at different temperatures from 8 °C to 88 °C with an interval temperature of 8 °C, and the results were obtained by performing classical time–temperature superposition shifts at a reference temperature of at 24 °C, deduced from the data in Figure 2 using horizontal shift factor a_T_ and without vertical shift. Arrhenius plot depicting the temperature dependence of the shift factors a_T_ used for generating master curves of DN−Zr^4+^−0.1 M−AP hydrogels (**c**) and DN−Zr^4+^−0.1 M−E hydrogels (**d**). The apparent activation energy values were calculated from the slope of the curves.

The dynamic mechanical study can be used to clarify the strength of the dynamic bonds within the hydrogel system [9]. Based on the above isothermal frequency sweep data collected over a wide range of temperatures, we constructed the master curve according to the time–temperature superposition (TTS) principle for DN−Zr^4+^−0.1 M−AP and DN−Zr^4+^−0.1 M−E hydrogels [44,45]. The reference temperature was set at 24 °C. For each gel, the frequency sweep curves were shifted horizontally with a temperature-dependent shift factor a_T_, and no vertical shift was performed. As shown in Figure 3a,b, both the DN−Zr^4+^−0.1 M−AP and DN−Zr^4+^−0.1 M−E hydrogels show well-superposed time–temperature master curves. The Arrhenius plot for the shift factor of the master curve, a_T_, is shown in Figure 3c for the DN−Zr^4+^−0.1 M−AP gels and Figure 3d for the DN−Zr^4+^−0.1 M−E hydrogels, from which the activation energy E_a_ can be extracted from the Arrhenius equation: *a*_T_ = *Ae^E^*^a/*RT*^, where R is the ideal gas constant and A is a constant. The *a*_T_-1/T plot gives an apparent activation energy (E_a_) of ≈80.8 kJ mol^−1^ for the DN−Zr^4+^−0.1 M−AP gels. This relatively low apparent activation energy (E_a_) is considered to be due to weakened metal coordinated bonds caused by the existence of excessive ions. Thus, over an ordinary time scale, these bonds have a relatively short relaxation time, which easily dissociates during deformation. Meanwhile, for the DN−Zr^4+^−0.1 M−E hydrogels, the plot of ln a_T_ verse 1/T shows very different slopes at low and high temperature regimes (Figure 3d). Based on this plot, two apparent activation energy values of 48.6 kJ/mol and 160.9 kJ/mol were obtained, indicating two kinds of physical bond with different bonding strengths. The differentiation of bonding strengths in the DN−Zr^4+^−0.1 M−E hydrogels could be attributed to the inhomogeneous redistribution of coordinated chains during the dialysis of excess ions from gels, confirmed in charge-balanced polyampholytes gels in previous publications [46]. For DN−Zr^4+^−0.1 M−AP gels, the gels show a relatively homogeneous structure in the presence of excess ions. When immersing the gels in DI water, the rapid dialysis of ions out of the gels leads to the redistribution of polymer chains into the polymer’s dense and dilute phases. Due to the high density of the polymer chain in the dense phase, the multivalent metal ions are more tightly coordinated with sulfonate, resulting in a larger bond energy, which means that more energy is required to break this coordination bond (i.e., a larger E_a_); conversely, in the dilute phase, the loose molecular chains make the metal coordination interaction weaker, leading to lower coordination strength (and a lower E_a_).

### 2.3. Large Deformation Mechanical Behaviors and Energy Dissipation Behaviors

**Figure 4:**
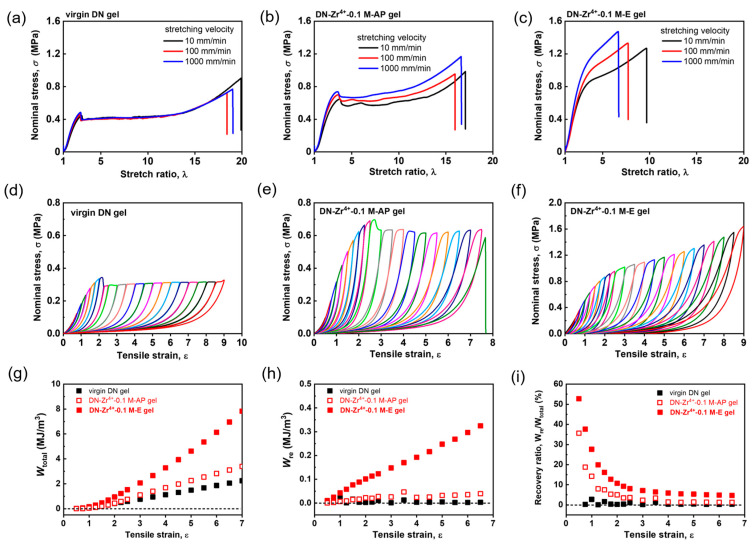
Tensile behaviors and cyclic tensile behaviors for various DN hydrogels with Zr^4+^ as metal ions for the metal coordination complex. (**a**) Rate-independent elastic tensile behaviors of the virgin DN hydrogels under different deformation rates ranging from 10 to 1000 mm/min. (**b**) Rate-dependent viscoelastic tensile behaviors of the DN−Zr^4+^−0.1 M−AP hydrogels under different deformation rates ranging from 10 to 1000 mm/min. (**c**) Rate-dependent viscoelastic tensile behaviors of the DN−Zr^4+^−0.1 M−E hydrogels under different deformation rates ranging from 10 to 1000 mm/min. (**d**–**f**) Sequential loading–unloading cycles for the virgin DN gels (**d**), DN−Zr^4+^−0.1 M−AP hydrogels (**e**), and DN−Zr^4+^−0.1 M−E hydrogels (**f**) at a deformation rate of 100 mm/min. The virgin DN hydrogels show completely irreversible hysteresis, while the rate-dependent viscoelastic DN−Zr^4+^−0.1 M−AP hydrogels and DN−Zr^4+^−0.1 M−E hydrogels show partially reversible hysteresis. The total mechanical hysteresis W_total_ (**g**), the reversible mechanical hysteresis W_re_ (**h**), and the recovery ratio W_re_/W_total_ (**i**) as functions of tensile strain for various DN hydrogels.

The above rheological results clarified the bond strength change during the gel fabrication. However, these rheological results can only be used to evaluate the small-strain dynamic mechanical behaviors of DN hydrogels. Next, we evaluate the large deformation mechanical behaviors of various DN hydrogels by performing tensile tests under different deformation rates ranging from 10 to 1000 mm/min, corresponding to a strain rate range of 0.014 to 1.4 s^−1^. Figure 4a–c shows the tensile curves for the virgin DN hydrogels as well as the Zr^4+^ ion-loaded DN−Zr^4+^−0.1 M−AP and DN−Zr^4+^−0.1 M−E hydrogels, respectively. With increasing strain rate over two orders from 0.014 to 1.4 s^−1^, the tensile curves of virgin DN hydrogels are perfectly overlapped, indicating the rate-independency at large deformation. This clearly shows that the virgin DN hydrogels consisting of two elastic polymer networks are purely elastic with negligible interactions, consistent with the small-strain rheological results shown in Figure 2a–c. Meanwhile, for the Zr^4+^ ion-loaded DN−Zr^4+^−0.1 M−AP and DN−Zr^4+^−0.1 M−E hydrogels, with increasing strain rate, the tensile curves no longer overlap with each other and seem to slightly deviate from each other in the small deformation region, and then largely deviated in the large deformation region. The observed strong rate-dependent large deformation mechanical behaviors should result from the formation of dynamic and reversible sulfonate–Zr^4+^ coordination interactions in the PAMPS first network, which is strong evidence that the gels are viscoelastic [9]. At a low strain rate, the characterization relaxation time is smaller than the strain rate, where the dynamic bonds are easy to relax, resulting in negligible contribution to strength; at a high strain rate, the dynamic bonds do not have enough time to relax, and thereby act as permanent chemical bonds during tensile deformation, resulting in pronounced increase in strength and deviation from that at low strain rate [47]. Other research has also reported that the dynamic nature of metal coordination interactions imparts strong rate-dependent large deformation mechanical performance to a Zr^4+^ ion-toughened PAMPS single network hydrogel [41] and Fe^3+^ ion-toughened P(AAm-co-AAc) single network hydrogel [48]. The effect of deformation rate on the mechanical behaviors of the hydrogels reported was mainly attributed to the increase in the bonding strength with the deformation rate [48]. As shown in Figure 3b,c, the DN−Zr^4+^−0.1 M−E hydrogels show much stronger deformation rate dependence than the DN−Zr^4+^−0.1 M−AP hydrogels. This is attributed to the excess of Zr^4+^ ions in DN−Zr^4+^−0.1 M−AP hydrogels weakening the sulfonate–Zr^4+^ coordination interactions in the first PAMPS network; after the water-equilibrating process, the excess of Zr^4+^ ions will be dialyzed out from the gel, helping the formation of strong sulfonate−Zr^4+^ coordination in the first PAMPS network, which is consistent with the results deduced from time–temperature superposition. 

Owing to the dynamic nature of the sulfonate–Zr^4+^ coordination, the Zr^4+^ ion-loaded DN hydrogels should have completely different internal fracture mechanisms compared to the virgin DN hydrogels. To examine the different internal fracture mechanisms, we perform cyclic tensile tests on various DN hydrogels to check their energy dissipation behaviors during the cyclic loading and unloading process. Figure 4d–f shows the sequential loading–unloading curves of the virgin DN hydrogels, DN−Zr^4+^−0.1 M−AP and DN−Zr^4+^−0.1 M−E hydrogels, respectively. As exhibited in Figure 4d, the virgin DN hydrogels only show irreversible hysteresis, similar to the results reported in previous studies [49,50]. For conventional DN hydrogels consisting of two chemically crosslinked elastic polymer networks, an abundance of covalent bonds in the densely crosslinked elastic first network sacrificially fractures to dissipate a large amount of energy upon deformation. Such covalent bonds cannot be recovered once bond scission occurs, resulting in irreversible mechanical hysteresis, as observed in Figure 4d. For the DN−Zr^4+^−0.1 M−AP and DN−Zr^4+^−0.1 M−E hydrogels (Figure 4e,f), in addition to the irreversible mechanical hysteresis, a certain amount of reversible mechanical hysteresis can also be observed between loading–unloading tensile cycles. The partially reversible mechanical hysteresis could be attributed to the dynamic nature of sulfonate–Zr^4+^ coordination, which can break and reform to some extent upon loading and unloading tensile cycles. Such results are consistent with the previous reports showing that when the first network contains noncovalent interactions as dynamic sacrificial bonds, both reversible and irreversible mechanical hysteresis appear in the cyclic tensile and compressive tests [11,32,50]. We have to mention here that the irreversible mechanical hysteresis observed for the DN−Zr^4+^−0.1 M−AP and DN−Zr^4+^−0.1 M−E hydrogels is dominant in the energy dissipation behaviors compared to the reversible part of mechanical hysteresis, meaning that the covalent bond scission still dominates the internal fracture mechanism for such Zr^4+^-loaded DN hydrogels upon deformation. In comparison to the DN−Zr^4+^−0.1 M−AP hydrogels, the DN−Zr^4+^−0.1 M−E hydrogels not only exhibit enhanced total mechanical hysteresis (the absolute energy dissipation density W_total_), but also show enhanced reversible hysteresis W_re_, the hysteresis recovery ratio W_re_/W_total_ (Figure 4g–i). This result also suggests that for the DN−Zr^4+^−0.1 M−AP hydrogels, the amount or the strength of sulfonate–Zr^4+^ coordination is weakened due to the excess amount of Zr^4+^ ions, while the DN−Zr^4+^−0.1 M−E hydrogels can form relatively strong robust sulfonate−Zr^4+^ coordination interactions in the first PAMPS network by removing the excess amount of Zr^4+^ ions through dialysis of the hydrogels after the water-equilibrating process.

**Figure 5:**
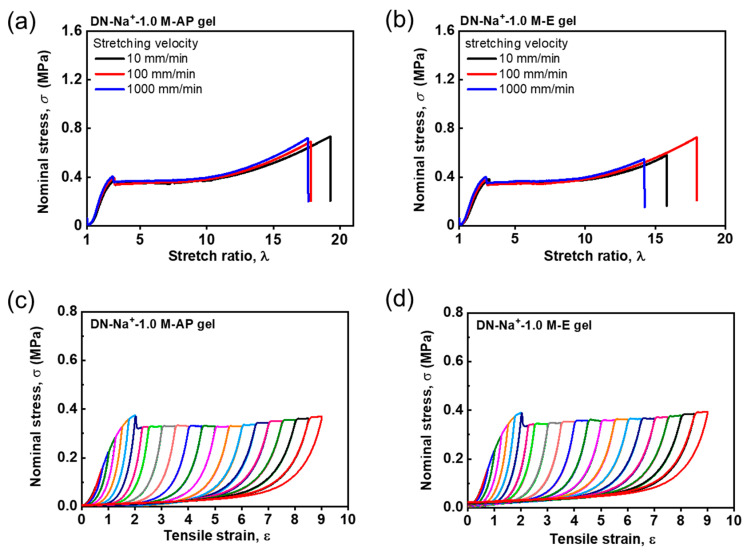
Tensile behaviors and cyclic tensile behaviors for various DN hydrogels with Na^+^ as metal ions. (**a**,**b**) Rate-independent tensile behaviors of the DN−Na^+^−1.0 M−AP (**a**) and DN−Na^+^−1.0 M−E (**b**) hydrogels under different deformation rates ranging from 10 to 1000 mm/min. (**c**,**d**) Sequential loading–unloading cycles for the DN−Na^+^−1.0 M−AP hydrogels (**c**) and DN−Na^+^−1.0 M−E hydrogels (**d**) at a deformation rate of 100 mm/min. The rate-independent elastic DN−Na^+^−1.0 M−AP and DN−Na^+^−1.0 M−E hydrogels show completely irreversible hysteresis.

**Figure 6:**
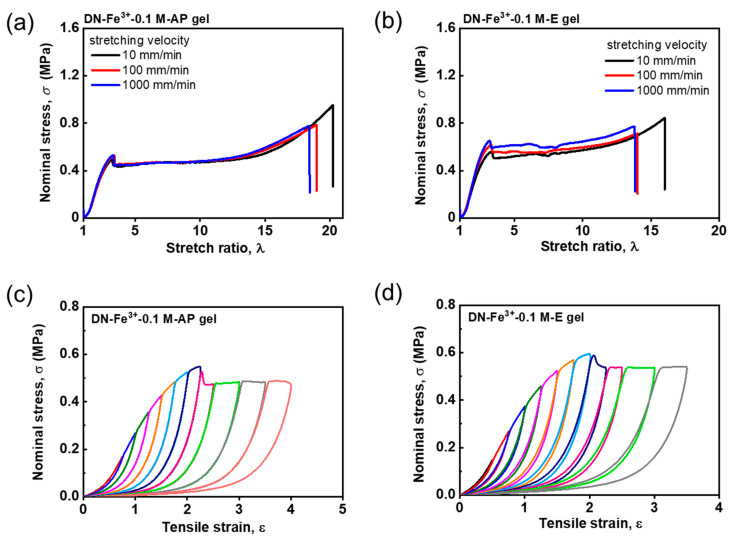
Tensile behaviors and cyclic tensile behaviors for various DN hydrogels coordinated with Fe^3+^ ions. (**a**,**b**) Rate-independent tensile behaviors of the DN−Fe^3+^−0.1 M−AP (**a**) and rate-dependent tensile behaviors of the DN−Fe^3+^−0.1 M−E (**b**) hydrogels under different deformation rates ranging from 10 to 1000 mm/min. (**c**,**d**) Sequential loading–unloading cycles for the DN−Fe^3+^−0.1 M−AP hydrogels (**c**) and DN−Fe^3+^−0.1 M−E hydrogels (**d**) at a deformation rate of 100 mm/min. The rate-independent elastic DN−Fe^3+^−0.1 M−AP hydrogels show irreversible hysteresis, while the rate-dependent viscoelastic DN−Fe^3+^−0.1 M−E hydrogels show partially reversible hysteresis owing to the metal coordination interactions.

To investigate the specificity of Zr^4+^ ions to form robust coordination with the sulfonate groups in the PAMPS first network, we further evaluate the tensile behaviors and cyclic tensile behaviors for various Na^+^ and Fe^3+^ ion-loaded DN hydrogels. Figure 5a,b shows the rate-independent tensile behaviors of the DN−Na^+^−1.0 M−AP and DN−Na^+^−1.0 M−E hydrogels, respectively. They are exactly similar to those of the virgin DN hydrogels shown in Figure 4a. The DN−Na^+^−1.0 M−AP and DN−Na^+^−1.0 M−E hydrogels also exhibit irreversible mechanical hysteresis in the loading–unloading cycles, similar to the virgin DN hydrogels (Figure 5c,d). These pure elastic mechanical behaviors suggest that Na^+^ ions cannot form interactions with the first network in the DN hydrogels because monovalent Na^+^ ions lack coordination ability. Figure 6a,b show the tensile behaviors of the Fe^3+^ ion-loaded DN−Fe^3+^−0.1 M−AP and DN−Fe^3+^−0.1 M−E hydrogels, respectively. Interestingly, the DN−Fe^3+^−0.1 M−AP hydrogels show rate-independent tensile behaviors, while the DN−Fe^3+^−0.1 M−E hydrogels demonstrate slightly rate-dependent tensile behaviors. Additionally, it is found that the DN−Fe^3+^−0.1 M−AP hydrogels show completely irreversible hysteresis, while the DN−Fe^3+^−0.1 M−E hydrogels show partially reversible hysteresis in Figure 6c,d. These results suggest that the DN−Fe^3+^−0.1 M−AP hydrogels have a pure elastic first network with negligible metal coordination interactions, while DN−Fe^3+^−0.1 M−E hydrogels have a slightly viscoelastic first network with weak metal coordination interactions. The distinct difference between the DN−Fe^3+^−0.1 M−AP hydrogels and the DN−Fe^3+^−0.1 M−E hydrogels indicates that weak sulfonate–Fe^3+^ coordination interactions can only be formed after getting the excess amount of Fe^3+^ dialyzed out of the hydrogels.

### 2.4. Rescaled Mechanical Behaviors

The highly crosslinked first network, which is also in its highly stretching state due to the osmotic pressure-induced swelling effect, dominates the mechanical performance of the DN hydrogels [50,51,52,53]. To have a clear and quantitative understanding of the effect of various ions on the mechanical performance of various ion-loaded DN hydrogels, we need to normalize the strand density and pre-stretch level of the first network in different systems relative to its as-prepared state in order to remove the effect of osmotic pressure-induced swelling. Here, we used the normalization method for DN material systems by rescaling the nominal stress (*σ*) and stretch ratio (λ) with the pre-stretch ratio of the first network (λ_s_) to the rescaled stress σλ_s_^2^ and the rescaled stretch ratio λλ_s_, respectively [50,52,53]. 

**Figure 7:**
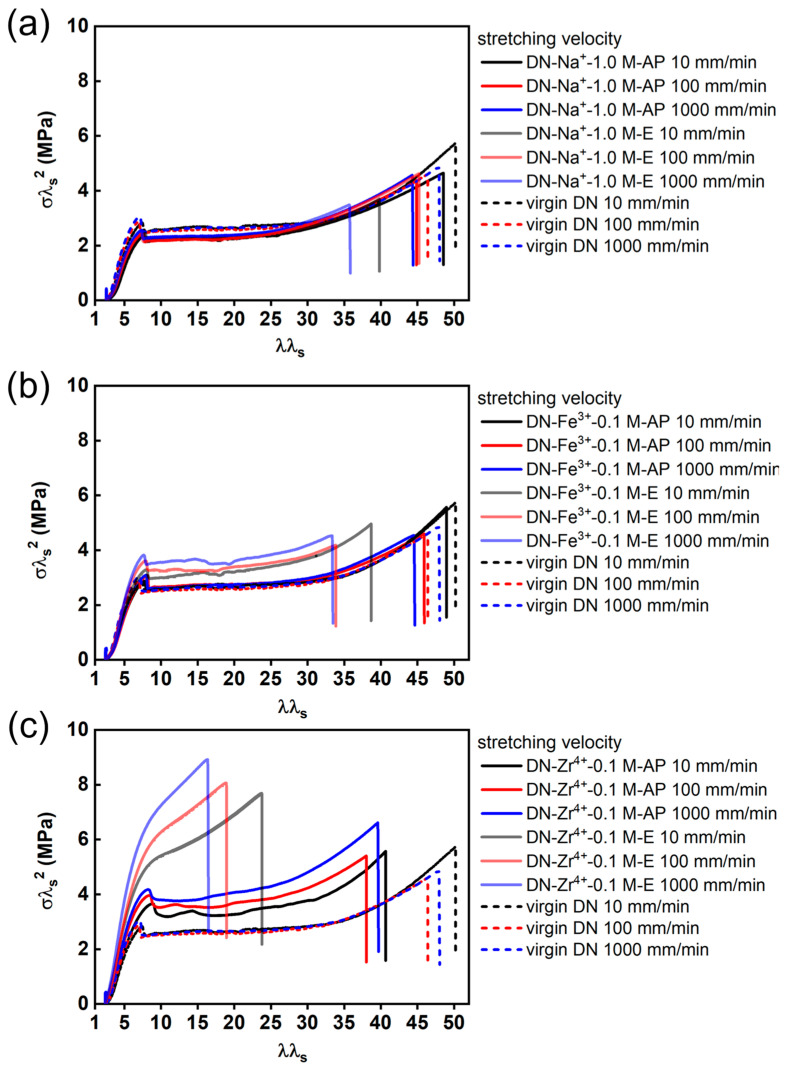
(**a**–**c**) The rescaled stress σλ_s_^2^–rescaled stretch ratio λλ_s_ curves for DN hydrogels coordinated with Na^+^ (**a**), Fe^3+^ (**b**), and Zr^4+^ (**c**) as metal ions under different deformation rates ranging from 10 to 1000 mm/min.

Figure 7 shows the rescaled stress σλ_s_^2^–rescaled stretch ratio λλ_s_ curves for various DN hydrogels loaded with Zr^4+^, Fe^3+,^ and Na^+^ as metal ions under different deformation rates ranging from 10 to 1000 mm/min in comparison to the virgin DN hydrogels. As shown in Figure 7a, these perfectly overlapped rescaled stress curves for the DN−Na^+^−1.0 M−AP hydrogels and DN−Na^+^−1.0 M−E hydrogels under different deformation rates indicate the negligible effect of Na^+^ ions due to the lack of coordination ability between monovalent Na^+^ ions and sulfonate groups in the PAMPS first network. The DN−Fe^3+^−0.1 M−AP hydrogels, as shown in Figure 6b, show perfectly overlapped rate-independent rescaled tensile curves with those of the virgin DN hydrogels; the Na^+^ ion-loaded DN hydrogels are similar, suggesting the negligible influence of Fe^3+^ ions in the DN−Fe^3+^−0.1 M−AP hydrogels. Given that Fe^3+^ ions have coordination ability with the sulfonate groups in the PAMPS first network, such negligible influence of Fe^3+^ ions in the DN−Fe^3+^−0.1 M−AP hydrogels can be explained by the fact that the excess amount of Fe^3+^ ions weakens the Fe^3+^-coordination interactions. Note that the ion concentration of Fe^3+^ ions in the solution (C_m_ = 0.1 M) is larger than the effective concentration of sulfonate functional groups (~0.05 M). As opposed to the DN−Fe^3+^−0.1 M−AP hydrogels, the water-equilibrated DN−Fe^3+^−0.1 M−E hydrogels show slightly enhanced stress with a slight rate dependency in the rescaled curves compared to these of virgin DN hydrogels (Figure 6b), suggesting that the formation of weak sulfonate–Fe^3+^ coordination interactions in the water-equilibrated DN−Fe^3+^−0.1 M−E hydrogels only contributes slightly to the mechanical enhancement of the DN hydrogels after the excess amount of Fe^3+^ has been dialyzed out of the hydrogels. 

For the Zr^4+^–coordinated DN hydrogels, as exhibited in Figure 7c, both the DN−Zr^4+^−0.1 M−AP hydrogels and the DN−Zr^4+^−0.1 M−E hydrogels deviate largely from the virgin DN hydrogels in the rescaled stress curves, indicating a strong rate dependency. The mechanical enhancement of Zr^4+^ ions is more significant in the DN−Zr^4+^−0.1 M−E hydrogels than in the DN−Zr^4+^−0.1 M−AP hydrogels. This result clearly shows that the Zr^4+^ ions in the DN−Zr^4+^−0.1 M−E hydrogels can form relatively robust sulfonate–Zr^4+^ coordination in the first PAMPS network, contributing to the mechanical enhancement. Meanwhile, for the DN−Zr^4+^−0.1 M−AP hydrogels, the enhancement is weakened due to an excess amount of Zr^4+^ ions. Although the mechanical enhancement of Zr^4+^ ions in the DN−Zr^4+^−0.1 M−AP hydrogels is comparatively weakened from that in the DN−Zr^4+^−0.1 M−AP hydrogels, the enhancement is more obvious than the Fe^3+^ ions in the DN−Fe^3+^−0.1 M−E hydrogels. This suggests that the bond strength of sulfonate–Zr^4+^ coordination is stronger than sulfonate–Fe^3+^ coordination interactions. This has also been verified in a previous report showing that the PAMPS hydrogels cannot be effectively toughened by Fe^3+^ ions but can be remarkably toughened by Zr^4+^ ions [41]. This specific effect of Zr^4+^ ions has been generally explained by the hard–soft acid–base theory [41,54]. Based on the hard–soft acid–base theory, the Zr^4+^ ions with four positive charges and a small radius of 0.72 Å belong to hard acids, which can accept non-bonding electron pairs [55]; the sulfonate groups can be seen as hard bases owing to the high electronegativity of the oxygen atom, serving as multidentate ligands to coordinate with Zr^4+^ ions. Moreover, the roughly spherical shape of the electron density of the sulfonate groups has more coordinative flexibility to form robust metal coordination bonds [56].

**Figure 8:**
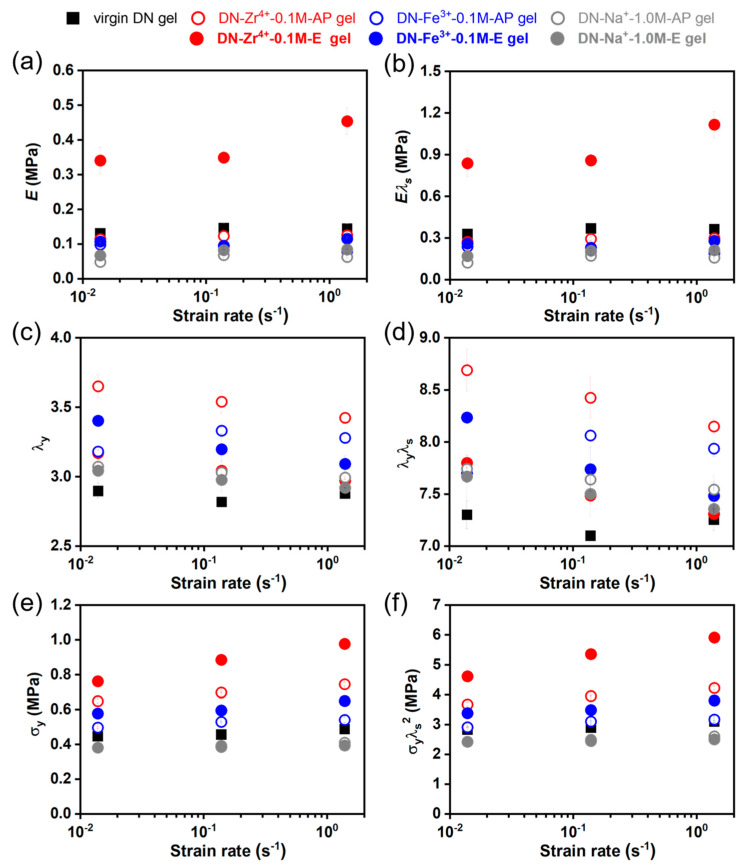
(**a**,**b**) Dependence of the Young’s modulus *E* (**a**) and the rescaled Young’s modulus *E*λ_s_ (**b**) on strain rate for various DN hydrogels. (**c**,**d**) Dependence of the yielding stretch ratio λ_y_ (**c**) and the rescaled yielding stretch ratio λ_y_λ_s_ (**d**) on strain rate for various DN hydrogels. (**e**,**f**) Dependence of the yielding stress σ_y_ (**c**) and the rescaled yielding stress σ_y_λ_s_^2^ (**d**) on strain rate for various DN hydrogels.

Next, we systematically discuss the effect of ion types on the Young’s modulus *E*, the yielding stretch ratio λ_y,_ and the yielding stress σ_y_. Figure 8a,b depicts the dependence of the Young’s modulus *E* and the rescaled Young’s modulus *E*λ_s_ on strain rate for various DN hydrogels. Except for the DN−Zr^4+^−0.1 M−E hydrogels, all series have a Young’s modulus *E* and a rescaled Young’s modulus *E*λ_s_ that are comparable with those of virgin DN hydrogels, and show almost no rate dependency. The DN−Zr^4+^−0.1 M−E hydrogels show a distinctively enhanced Young’s modulus *E* and rescaled Young’s modulus *E*λ_s_, and exhibit a slightly increasing tendency with the strain rate. The robust sulfonate–Zr^4+^ coordination stiffens the polymer network by increasing the effective crosslinking density, because these strong dynamic bonds can serve as temporary crosslinks to restrict the movements of polymer strands.

As shown in Figure 7, all series of hydrogels except for the DN−Zr^4+^−0.1 M−E hydrogels exhibit the typical tensile behaviors accompanied by a distinct stress-yielding, remarkable necking, and subsequent strain-hardening phenomena [10]. It has already been revealed in the toughening mechanisms of DN hydrogels that the yielding corresponds to the catastrophic fracture of the brittle first network into discontinuous fragments [51]. Thus, the rescaled yielding stretch ratio λ_y_λ_s_ possibly represents the stretching limit of the brittle first network strands [51]. Although the DN−Zr^4+^−0.1 M−E hydrogels do not show apparent stress-yielding behavior, we also denote the transition point between two stress-regions, showing contrasting slopes as the yielding point. Figure 8c,d depicts the dependence of the yielding stretch ratio λ_y_ and the rescaled yielding stretch ratio λ_y_λ_s_ on strain rate. Interestingly, we find that the DN−Zr^4+^−0.1 M−AP hydrogels with excess amounts of Zr^4+^ ions show a much more enhanced rescaled yielding stretch ratio λ_y_λ_s_ compared to other DNs, including the virgin DN hydrogels as well as the water-equilibrated DN–Zr^4+^–0.1 M–E hydrogels. This suggests that the first network in the DN−Zr^4+^−0.1 M−AP hydrogels has a much improved stretching limit of the brittle first network strands. The brittle first network strands have a wide distribution of strand lengths due to their inhomogeneity; the short strands in first network would break at a small stretching limit, while the long strands would break at a large stretching limit. This improvement of stretching limit by excessive Zr^4+^ ions in the DN−Zr^4+^−0.1 M−AP hydrogels may be explained by the fact that the sulfonate–Zr^4+^ coordination interactions may homogenize the distribution of strand lengths by adding self-adjustable dynamic crosslinks in the inhomogeneous first network strands; this reduces the effective fracture of these short strands. This homogenization seems to be effective only when the metal coordination is neither strong enough nor weak enough, which is also observed for the DN−Fe^3+^−0.1 M−E hydrogels, having relatively weak sulfonate–Fe^3+^ coordination interactions. Moreover, with increasing strain rate, the DN−Zr^4+^−0.1 M−AP hydrogels, DN−Zr^4+^−0.1 M−E hydrogels, and DN−Fe^3+^−0.1 M−E hydrogels show a decreasing tendency. This is reasonable because the dynamic nature of metal coordination determines that the bond strength increases with the deformation rate [41,48].

**Figure 9:**
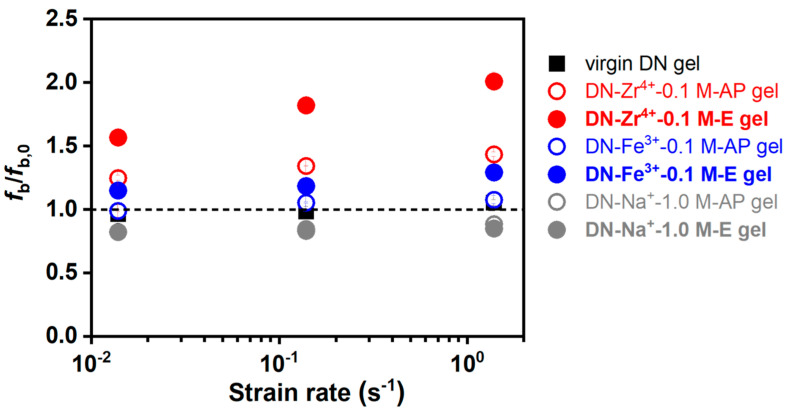
The ratio of breaking force of first network strands *f*_b_/*f*_b,0_ for various DN hydrogels relative to the virgin DN gels. The *f*_b_ and *f*_b,0_ represent the breaking force of first network in various DN hydrogels and the virgin DN gels, respectively.

We also check the dependence of the yielding stress σ_y_ and the rescaled yielding stress σ_y_λ_s_^2^ on strain rate for various DN hydrogels in Figure 8e,f. The yielding stress is typically interpreted as the total load sustained by the first network strands when catastrophic fracture occurs in first network. The DN−Zr^4+^−0.1 M−AP hydrogels, DN−Zr^4+^−0.1 M−E hydrogels, and DN−Fe^3+^−0.1 M−E hydrogels show remarkably enhanced yielding stress σ_y_ and rescaled yielding stress σ_y_λ_s_^2^ compared to the virgin DN hydrogels. This result suggests that the metal-coordination interactions can significantly increase the total load sustained by the first network strands when catastrophic fracture occurs in first network. Notably, the bond strength of metal coordination has the following order: DN−Zr^4+^−0.1 M−E > DN−Zr^4+^−0.1 M−AP > DN−Fe^3+^−0.1 M−E > DN−Fe^3+^−0.1 M−AP hydrogels. In addition, with increasing the strain rate, the DN−Zr^4+^−0.1 M−AP hydrogels, DN−Zr^4+^−0.1 M−E hydrogels, and DN−Fe^3+^−0.1 M−E hydrogels show an increasing tendency in the yielding stress σ_y_ and rescaled yielding stress σ_y_λ_s_^2^. This can be ascribed to the increased bond strength with the deformation rate. The rescaled yielding stress σ_y_λ_s_^2^ is proportional to the area density of the first network at the reference state (*v*_1st_^2/3^) times the bond-breaking force per strand (*f*_b_), $\sigma_{y}\lambda_{s}{}^{2}\propto v_{1st}{}^{2/3}f_{b}$ [57]. Thus, we have $\frac{\sigma_{y}\lambda_{s}{}^{2}}{{\left(\sigma_{y}\lambda_{s}{}^{2}\right)}_{virgin}}=\frac{f_{b}}{f_{b,0}},$ because all DN hydrogels are prepared with the same PAMPS network (the area density of the first network at the reference state (*v*_1st_^2/3^) should be constant), where the *f*_b_ and *f*_b,0_ represent the breaking force of first network in the ion-coordinated DN hydrogels and virgin DN gels, respectively. By normalizing all the rescaled yielding stress σ_y_λ_s_^2^ with (σ_y_λ_s_^2^)*_virgin_*, we can have a quantitative understanding of how much the breaking force of first network strands would be enhanced. Figure 9 depicts the ratio of breaking force of first network strands *f*_b_/*f*_b,0_ for various DN hydrogels relative to the virgin DN gels. The result suggests that the breaking force of first network strands can be significantly enhanced to 1.5 to 2-folds in the DN−Zr^4+^−0.1 M−E hydrogels, owing to the strong sulfonate–Zr^4+^ coordination interactions compared to the virgin DN gels.

**Figure 10:**
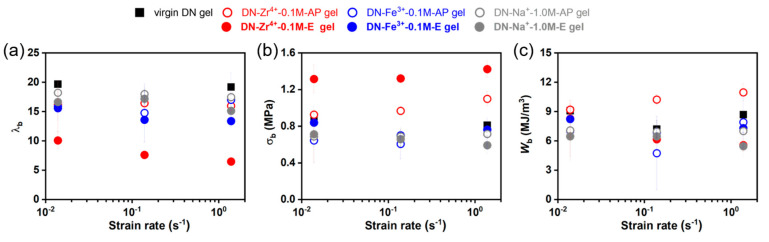
(**a**–**c**) Dependence of the stretch ratio at break λ_b_ (**a**), the stress at break σ_b_ (**b**), and work of extension *W*_b_ (**c**) on strain rate for various DN hydrogels.

The huge difference in the strength and stretchability of the brittle PAMPS first network and the stretchable PAAm second network dominates the load-transfer mechanisms upon deformation, thereby affecting the fracture behaviors at break of DN gels [43,58]; the maximum stretch ratio λ_b_ and fracture stress at break σ_b_ should reflect the relative strength/stretchability of the two networks. We next discuss the mechanical behaviors at break for various DN hydrogels. Figure 10 depicts the λ_b_, σ_b,_ and work of extension *W*_b_. All series of DN hydrogels have the same stretchable PAAm network which is hardly affected by the different ions; thus, the λ_b_ and σ_b_ of the PAMPS first network for different series of DN hydrogels should be comparable. Interestingly, the DN−Zr^4+^−0.1 M−E hydrogels show a significantly reduced maximum stretch ratio at break λ_b_, but remarkably enhanced fracture stress at break σ_b_, as shown in Figure 10a,b. This result indicates that the sulfonate–Zr^4+^ coordination formed in the DN−Zr^4+^−0.1 M−E hydrogels increases the relative strength of first network while decreasing its relative stretchability. On the other hand, the DN−Zr^4+^−0.1 M−AP hydrogels demonstrate slightly enhanced fracture stress at break σ_b_, accompanied by the hardly reduced maximum stretch ratio at break λ_b_ compared to the virgin DN hydrogels. This also suggests that the relatively weak sulfonate–Zr^4+^ coordination interactions will slightly increase the relative strength of first network without sacrificing its relative stretchability, thereby effectively increasing the work of extension *W*_b_ (even enhanced compared to the DN−Zr^4+^−0.1 M−E hydrogels). The work of extension *W*_b_ can also be used to characterize the toughness of the soft materials; the slightly increased work of extension *W*_b_ of the DN−Zr^4+^−0.1 M−AP hydrogels indicates that the bond strength can be neither strong nor weak to enhance the overall toughness upon deformation.

## 3. Conclusions

In summary, we presented a facile strategy for transforming the rate-independent elastic DN hydrogels to rate-dependent viscoelastic DN hydrogels via metal-coordinated with sulfonate functional groups in the PAMPS first network. The effect of metal coordination on the small strain rheology, yielding behaviors, and large strain hysteresis was systematically studied. Our results show that the viscoelasticization of DN gels can be achieved via metal ions (Fe^3+^, Zr^4+^) which have a coordinated ability with sulfonate. In the DN−Zr^4+^−0.1 M−AP gels, the excessive ions impart a metal coordination bond with homogeneous bong strength (Ea: 80.8 kJ/mol). Meanwhile, the dialysis process differentiates the bond strength into two values (48.6 kJ/mol and 160.9 kJ/mol), which is possibly attributed to the inhomogeneous redistribution of coordinated chains. In our observation time scale, we found that stronger bond strength leads to a larger yielding strength and a larger hysteresis ratio, which originates from the enhancement strength of first network strand via the metal coordination formed in first network chain. Based on quantitative analysis, we deduced that the breaking force of first network strands could be increased by 1.5 to 2-folds in the DN−Zr^4+^−0.1 M−E hydrogels compared to the virgin DN gels. The maximum toughness of the metal-coordinated DN hydrogels is achieved with a moderate dynamic bond strength, which is a synergy of increasing breaking force of polymer chains to dissipate energy, and keeping deformability due to structural homogenization.

## 4. Materials and Methods

### 4.1. Materials 

The 2-acrylamido-2-methylpropanesulfonic acid, acrylamide (AAm, purity ≥ 99.8%), *N*,*N*′-methylenebis(acrylamide) (MBAA, purity ≥ 99.0%), and *α*-ketoglutaric acid (*α*-keto, purity: 99%) were purchased from Shanghai Aladdin Bio-Chem Technology Co., Ltd. (Shanghai, China) and used as received. DI water (resistivity: 18.3 MΩ·cm) was used in all experiments.

### 4.2. Synthesis of the Virgin PAMPS/PAAm DN Hydrogels 

The poly(2-acrylamido-2-methylpropanesulfonic acid)/polyacrylamide (PAMPS/PAAm) DN hydrogels were synthesized by a two-step sequential network formation technique following the literature [43]. The first PAMPS network of the DN hydrogels was synthesized from an aqueous solution of 1.0 M AMPS containing 3 mol% crosslinking agent, MBAA, and 1 mol% initiator, *α*-keto. The solution was purged in an argon atmosphere to remove dissolved oxygen and then poured into a reaction cell consisting of a pair of glass plates with 0.5 mm spacing. The reaction cell was irradiated with UV light (365 nm) for 8 h. These gels (first network) were then immersed in an aqueous solution of 2.0 M AAm, containing 0.01 mol% MBAA and 0.01 mol% *α*-keto, for one day until swelling equilibrium was reached. The polymerization was performed again by 365 nm UV irradiation for 8 h. The as-prepared DN gels were then immersed in pure water to reach equilibrium to obtain the virgin DN gels for further experiments.

### 4.3. Preparation of the “DN−M^n+^−C_m_−AP” and “DN−M^n+^−C_m_−E” Hydrogels 

The metal ion-loaded DN hydrogels were prepared first through immersion in metal–ion solutions and subsequently through an equilibrium approach. For simplification, the as-prepared DN hydrogels were first immersed in a metal–ion solution (in the as-prepared state) and subsequently transferred to DI water for equilibrium. For the sulfonate−Zr coordination, the reported paper shows that the atomic ratio of S to Zr is about 0.5, suggesting that one Zr cluster coordinated with two sulfonate groups [41]. For simplicity, the Zr^4+^ ion is used to show the coordination with sulfonate groups. For the sulfonate−Fe coordination, the Fe^3+^ ion can form with sulfonate in mono-, bis-, and tris-complex [42]; here, we use the simple notation “Fe^3+^−sulfonate” to represent the coordination bonds. Here, the DN hydrogels balanced in metal–ion solution and DI water were denoted as “DN−M^n+^−C_m_−AP” and “DN−M^n+^−C_m_−E” hydrogels, respectively, where M*^n^*^+^ represents Zr^4+^, Fe^3+^ and Na^+^ ions, and C_m_ (M) represents the metal–ion concentration in the solution. Typically, to prepare the DN−Zr^4+^−0.1 M−AP hydrogels, the virgin DN hydrogels were first soaked in 200 mL zirconium (IV) chloride (ZrCl_4_) solution (C_m_ = 0.1 M) at ambient temperature for one week to reach a swelling equilibrium in the solution. The obtained DN−Zr^4+^−0.1 M−AP hydrogels were then immersed in adequate DI water for at least one week to remove the excessive ions dialyzed from the hydrogels and to reach an equilibrium state in water (DN−Zr^4+^−0.1 M−E hydrogels). DI water was changed twice a day to ensure a sufficient dialysis process. 

### 4.4. Rheological Test 

Dynamic rheological tests were performed with an ARES rheometer (Advanced Rheometric Expansion System, Rheometric Scientific Inc.). A disk-shaped sample with a diameter of 15.0 mm was fixed between aluminum plates. The various DN hydrogel samples were surrounded by liquid paraffin during measurement to prevent water evaporation and ion diffusion from the DN hydrogels. Before each measurement, the DN hydrogel samples were held at the set temperature for 300 s to reach the temperature equilibrium. For the DN gels coordinated with Zr^4+^, the rheological measurements were performed over a frequency range of 0.1–100 rad/s with a shear strain of 0.2% at a temperature range of 8−88 °C. The data were processed into master curves of storage modulus *G′*, loss modulus *G″*, and loss factor tan δ via time−temperature superposition (TTS) shifts at a reference temperature of 24 °C. Meanwhile, for the virgin DN gels and gels coordinated with Fe^3+^ and Na^+^, the frequency sweep was only performed at reference temperature due to their weak temperature dependence of rheological behaviors. 

### 4.5. Tensile Test 

The tensile mechanical properties of various DN hydrogels were measured with a commercial test machine in the air (4466, Instron Instruments, Inc., Norwood, MA, USA). To prevent hydrogel dehydration, a humidifier was used during the tests. The samples were cut into dumbbell shapes standardized as JISK6251-7 size (gauge length 12 mm, width 2 mm) with a gel cutting machine. The nominal stress σ-stretch ratio *λ* curves were recorded while the sample gels were stretched at different constant velocities of 10, 100, and 1000 mm/min (corresponding to strain rates of 0.014, 0.14, and 1.4 s^−1^). The Young’s modulus, E, was calculated from the initial slope of the stress–strain curves at the stain within 10%. The work of tension of the samples during the tests, *W*_t_, was calculated by integrating the area under the stress–strain curves by the equation: $\int_{0}^{\lambda_{b}}\sigma d\lambda$, where σ and λ were the stress and stretching ratio, respectively, and $\lambda_{b}$ was the stretching ratio at breaking.

### 4.6. Cyclic Tensile Test

Cyclic tensile tests were performed using the same experimental setup as the tensile tests. The samples were stretched to a pre-set stretch ratio of 50%, 75%, 100%, 125%, 150%, 175%, 200%, 225%, 250%, 300%, 350%, 400%, 450%, 500%, 550%, 600%, 650%, 700%, 750%, 800%, 850%, and 900%, respectively, followed by unloading at the same strain rate, and the sequential loading−unloading cycles with increased stretch ratios were performed without waiting time. The stretching velocity was fixed at 100 mm/min.

## Data Availability

The data presented in this study are available on request from the corresponding author.

## Supplementary Materials

The following supporting information can be downloaded at: https://www.mdpi.com/article/10.3390/gels9020145/s1, Figure S1: Frequency dependence of (a) storage moduli G′, (b) loss moduli G″ and (c) loss factor tan δ for DN−Na^+^−1.0 M−AP and DN−Na^+^−1.0 M−E hydrogels.
