# Supplementary material for: Metal-Coordinated Dynamics and Viscoelastic Properties of Double-Network Hydrogels
Source: Gels. 2023 Feb 9;9(2):145. doi: 10.3390/gels9020145 (PMC9956398; doi:10.3390/gels9020145)
Supplement: Supplementary file 1 [file gels-09-00145-s001.zip › gels-2192975-supplementary.pdf]

Supporting Information for

Metal-Coordinated Dynamics and Viscoelastic Properties of Double-Network Hydrogels

Shilei Zhu [1,†], Yan Wang [2,†], Zhe Wang [2], Lin Chen [2], Fengbo Zhu [2,3], Yanan Ye [2,3,*], Yong Zheng [4,*], Wenwen Yu [2,3] and Qiang Zheng [2,5]

[1] College of Physics, Taiyuan University of Technology, Taiyuan 030024, China
[2] College of Materials Science & Engineering, Taiyuan University of Technology, Taiyuan 030024, China
[3] Shanxi-Zheda Institute of Advanced Materials and Chemical Engineering, Taiyuan 030024, China
[4] Institute for Chemical Reaction Design and Discovery, Hokkaido University, Sapporo 001-0021, Japan
[5] Ministry of Education Key Laboratory of Macromolecular Synthesis and Functionalization, Department of Polymer Science and Engineering, Zhejiang University, Hangzhou 310027, China
* Correspondence: yeyanan@tyut.edu.cn (Y.Y.), zhengyong@sci.hokudai.ac.jp (Y.Z.)
† These authors contributed equally to this work.

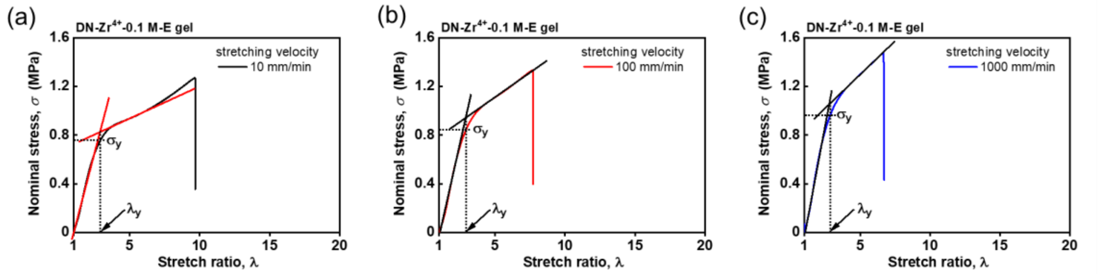

Figure S1. Determination of the yielding stretch ratio λ_y and the yielding stress σ_y for the DN-Zr^{4+}-0.1 M-E hydrogels without obvious yielding.
